# Patient health literacy and cognitive impairment surveys highlight barriers to patient-provider communication

**DOI:** 10.3389/frhs.2026.1727451

**Published:** 2026-02-13

**Authors:** Star Okolie, Neela Batthula, Anjana Shah, Alana Christie, Philippe Zimmern

**Affiliations:** Department of Urology, UT Southwestern Medical Center, Dallas, TX, United States

**Keywords:** cognitive impairment, health communication, health literacy, physician perception, urology

## Abstract

**Introduction:**

Providers may underestimate patient health literacy, and patients may not understand commonly used medical terms. Undiagnosed cognitive impairment among Urogynecology and Reconstructive Pelvic Surgery (URPS) patients may further hinder communication. We assessed communication barriers between URPS clinic patients and their providers.

**Methods:**

Following IRB approval, women aged 18–80 years attending outpatient visits were invited to participate. Non-English speakers and those with diagnosed cognitive impairment were excluded. Participants completed a health literacy measure (REALM-SF), a validated cognitive impairment screen (STMS questionnaire), and an 8th-grade level URPS Lexicon of ten medical terms. Providers, blinded to questionnaire results, then answered questions about their perception of the patient's health literacy and cognitive status.

**Results:**

From June to December 2024, 157 patients were invited to participate (9 declined). Of the 59 who scored in at least the mild impairment range (29–33) on the STMS, only 2 were noted by the provider as cognitively impaired. All words were correctly identified by 100 patients (68%), and at least 8 words by 82%. “Vagina” was the most commonly misdefined, followed by “bowel,” “pelvis,” and “urethra.” Providers identified 7 patients as having low health literacy, 4 of whom answered 6 or fewer words correctly, while 3 answered all 10 words correctly. Patients with lower health literacy had significantly lower STMS scores as compared to the patients with high-school level health literacy (*p* = 0.036).

**Conclusions:**

Discrepancies between provider assessments and screening results highlight the need for improved recognition of cognitive impairment and health literacy to enhance communication and patient care.

## Introduction

Although health literacy can have a variety of definitions, in this study it was defined as the extent to which patients can ascertain, understand, and discuss information about their health to make informed decisions on their healthcare (1). In any field of medicine, it is important for patients to have an adequate level of health literacy for quality shared decision-making with the provider. Disparate health outcomes that have resulted from limited health literacy have been documented in both cancer care for urology patients and in the realms of benign urology, as it impacts the quality of shared decision-making between patients and providers (2, 3). Previous studies in the field of female urology have found that adequate health literacy improved quality of life for patients with urinary incontinence and may be beneficial to address in education among nurses (2).

While there is some ambiguity in defining mild cognitive impairment, *Clinics in Geriatric Medicine* has defined it as an intermediate stage between memory impairment as a result of normal aging and dementia (4). Research has demonstrated a a prevalence of cognitive impairment among elderly urogynecologic patients and an association between urinary incontinence and cognitive health (5, 6). Considering the substantial prevalence of mild cognitive impairment in the older adult urology patient population, it is important for providers to understand how it may present, and how best to facilitate shared decision-making for patients with mild cognitive impairment (7). This is also imperative for patient quality of life, as mild cognitive impairment may increase patient risk for developing depressive symptoms and reduced quality of life in the context of pelvic floor disorders (8). It is not only vital for healthcare providers to recognize when a patient may have cognitive impairment or reduced health literacy, but also, it is crucial to develop ways to mitigate the negative impact cognitive impairment and reduced health literacy may have on patient health outcomes.

The existing limited literature on patient health literacy and cognitive impairment in urology mainly focuses on documenting prevalence. This gap in knowledge and the overlapping nature of health literacy and cognitive impairment warranted a joint assessment of both domains. There is a lack of qualitative data on patient health literacy and cognitive impairment in tertiary care urology clinics and how that compares to the provider's perception.

This study sought to assess barriers in provider-patient communication, including potential cognitive impairment or reduced health literacy. Additionally, discrepancies revealed between patient cognitive status and/or health literacy and the provider's perception could uncover areas of education to aid in shared clinical decision-making with patients from these groups.

## Materials and methods

### Study design

This was a prospective, blinded study using standardized measures for patient health literacy and cognitive status.

### Participants

Following Institutional Review Board (IRB) approval, female patients aged 18–80 years attending a tertiary care URPS specialized clinic who were new or returning to the URPS provider were invited to participate. Non-English speakers, patients under 18 years and over 80 years of age, those with a cognitive impairment diagnosis, and those who had previously been seen by a URPS physician in a different practice were excluded. Enrolled patients who visited more than once during the study period were only assessed at the time of enrollment. All patients were interviewed for this study before their visit with the URPS provider.

### Data collection

After patient consent, patient demographic information was collected, including date of birth, race/ethnicity, highest level of education completed, and total annual household income. The patient was then asked to respond to three standardized questionnaires consecutively, which were administered by the same research assistant for consistency. The Rapid Estimate of Adult Literacy in Medicine—Short Form (REALM-SF) and the Short Test of Mental Status (STMS) are well-established and validated measures that do not require specific training to be administered (9). Although it has not yet been a validated instrument, the Lexicon was developed by consensus among URPS faculty to reflect medical terms commonly used during routine clinic visits and patient counseling, with definitions mirroring those provided in standard patient education materials developed by established URPS societies. This was presented to patients in a single sheet and required matching commonly employed words in the URPS field to their respective (listed) definition.

Patients were provided REALM-SF sheet and instructed to read the words aloud if they recognized the word (9). A sum of the total words recognized and said aloud was totaled at the bottom of the sheet (out of a total of seven). A score of 0 indicated an education level of third grade and below, a score of 1–3 indicated a fourth-to-sixth grade level, a score of 4–6 indicated a seventh-to-eighth grade level, and a 7 out of 7 indicated a high-school level education (9).

After completing the REALM-SF, the patient then underwent a cognitive screen using the Short Test of Mental Status (STMS) (10). Patients were tested on orientation, attention, immediate recall, calculation, abstraction, construction, copying images, information, and delayed recall. The total score was summed from the points in each section of the STMS out of 38. A score below 29 indicated dementia, a score of 29–33 indicated mild cognitive impairment, and a score above 33 indicated no cognitive impairment (10).

After completing the STMS, the patient was then given a vocabulary worksheet containing words in the URPS Lexicon and definitions; patients were to match the correct word to its definition. After patient completion of the worksheet, the total correct words and the missed words were recorded. After the patient completed a scheduled visit with the provider, the provider, without knowledge of how the patient responded on any of the questionnaires, then answered two “yes-or-no” questions regarding whether the patient had an adequate level of health literacy and whether the patient had cognitive impairment. Provider assessments were intentionally limited to binary responses to reflect routine clinical impressions formed during standard visits, and were performed with the knowledge that their responses were being studied. All three questionnaires were administered by a research assistant who remained neutral and did not intervene in the answers to any of these questionnaires.

### Statistical methods

Descriptive statistics were provided as medians and interquartile ranges (IQR) for continuous measures, and as frequencies and percentages for categorical measures. Differences in age between STMS groups were tested using unpaired Student *t*-tests. Chi-square tests were used to assess for any associations between the categorical questionnaire responses between patients coming to URPS for their first visit vs. returning URPS patients, and unpaired Student *t*-tests were used for differences in continuous responses between those groups. The association between STMS score and REALM-SF group was tested using an unpaired Student *t*-test; the STMS score and URPS Lexicon group was tested using generalized linear modeling (GLM). A Chi-square test was used to analyze the association between REALM-SF group and URPS Lexicon group. All analyses were completed at the 0.05 significance level without adjustments for multiple comparisons using SAS 9.4 (SAS Institute Inc., Cary NC).

### Ethical considerations

This study was approved by the Institutional Review Board. For eligible patients, the process of data collection (collecting demographic information, completing the REALM-SF, STMS, and lLexicon vocabulary worksheet) was explained, and each section of the consent form was reviewed with the patient. The patient was then asked whether they would like to participate in the study and assured that the information in this study would not be shared with other providers or used for any purpose outside those of this project. Patient consent had no bearing on the patient's scheduled visit and patients were able to decline if they did not want to participate. Declining to participate had no impact on their URPS care.

## Results

### Participant characteristics

Demographic information was collected and is reported in Table 1. Of the 157 consecutive patients invited to participate, 9 patients declined due to reasons including time constraints, status as a physician, not bringing reading glasses, not fluently English-speaking, and personal discomfort with the cognitive status exam. The median age of participants was 66 years. Of those studied, 37.8% (*n* = 56) attended their first visit at the URPS clinic. Most patients identified as White (75.0%, *n* = 111), and a majority of patients had completed a college education (57.4%, *n* = 85). Of the patients who disclosed their annual household income, 47.3% (*n* = 70) had an annual household income over $100,000.

**Table 1:** Patient characteristics.

Characteristic	*N* (%) or Median (IQR) *N* = 148
Median age, years	66 (52.2–73.7)
1st URPS visit	56 (37.8%)
Race/ethnicity	
Black	20 (13.5%)
Hispanic	4 (2.7%)
Asian	4 (2.7%)
White	111 (75.0%)
Other	9 (6.1%)
Education	
Less than high school	2 (1.4%)
High school	31 (20.9%)
College	85 (57.4%)
Post-graduate	30 (20.3%)
Income	
<50K	34 (23.0%)
50K–100K	35 (23.6%)
>100K	70 (47.3%)
Declined	9 (6.1%)

### Patient results

Results from the Lexicon vocabulary sheet, REALM-SF, and STMS are listed in Table 2. Most patients scored a 7 out of 7 on the REALM-SF (94.6%, *n* = 140). The median STMS score was 34 out of 38. A majority of patients scored in the “no impairment” range, scoring over 33 out of 38 (60.1%, *n* = 89). There were 100 patients (67%) who answered all ten words in the Lexicon correctly, while 121 (82%) answered at least eight words correctly. The most missed word was “vagina” (36 patients), followed by “bowel” (22 patients), “pelvis” (21 patients), and “urethra” (21 patients). Time spent on the three questionnaires was 10–15 min, with the STMS taking the longest to administer. There was no significant difference in the age distribution between the STMS groups (*p* = 0.5).

**Table 2:** Questionnaire results.

Parameter	*N* (%) or Median (IQR) *N* = 148
Median Lexicon words correct	10 (8–10)
0–4	6 (4.1%)
5–7	21 (14.2%)
8–10	121 (81.8%)
Median REALM-SF score	7 (7–7)
4	1 (0.7%)
5	2 (1.4%)
6	5 (3.4%)
7	140 (94.6%)
Median STMS total score	34 (32–36)
STMS Score group	
No impairment (>33)	89 (60.1%)
Mild impairment (29–33)	45 (30.4%)
Dementia (<29)	14 (9.5%)

### Provider assessment

In the 89 patients with no impairment according to their STMS score, none were noted to have cognitive impairment by the provider. Out of the 59 patients who had at least mild impairment according to their STMS score, only two were noted by the provider as having cognitive impairment. The remaining 97% of patients with screening-identified cognitive impairment were perceived by providers as cognitively intact during the clinical encounter. Seven patients were thought by the provider to have an inadequate level of health literacy, of whom, six scored 7 out of 7 on the REALM-SF questionnaire, and one patient scored a 6. Four of the seven patients who providers assessed as having low health literacy answered six or fewer words correct on the Lexicon vocabulary sheet, with the other three answering all 10 correctly. Of note, five of these seven patients had scored in the cognitive impairment range on the STMS.

### New vs. returning patients

Results from analysis comparing patients at their first URPS clinic vs. returning patients are presented in Table 3. The median age for returning patients was 68 years, while the median age for new patients was 57.4 years (*p* = 0.0008). There was no significant difference found in the REALM-SF and the Lexicon scores between returning and new patients. The median STMS score was 34 for returning patients and 35 for new patients (*p* = 0.0041). Among the returning patients, 50 scored in the no impairment range; among the new patients, 39 scored in the no impairment range (*p* = 0.080).

**Table 3:** Comparison of questionnaire scores from patients at their first URPS visit vs. returning URPS patients.

Parameter	First URPS visit (*n* = 56)	Returning URPS patients (*n* = 92)	*P*
Median age	57.4 (46.2–71.3)	68.0 (56.0–75.9)	0.0008
REALM score			
4	0 (0)	1 (1.1)	0.7
5	1 (1.8)	1 (1.1)	
6	1 (1.8)	4 (4.4)	
7	54 (96.4)	86 (93.5)	
STMS			
Median total score	35 (33–36)	34 (31–35)	0.0041
No impairment	39 (69.6)	50 (54.4)	0.080
Mild impairment	15 (26.8)	30 (32.6)	
Dementia	2 (3.6)	12 (13.0)	
Lexicon			
Median % correct	100 (90–100)	100 (80–100)	0.5
0–4 correct	1 (1.8)	5 (5.4)	0.17
5–7 correct	5 (8.9)	16 (17.4)	
8–10 correct	50 (89.3)	71 (77.2)	

### Correlation of health literacy and cognitive ability

**Figure 1:**
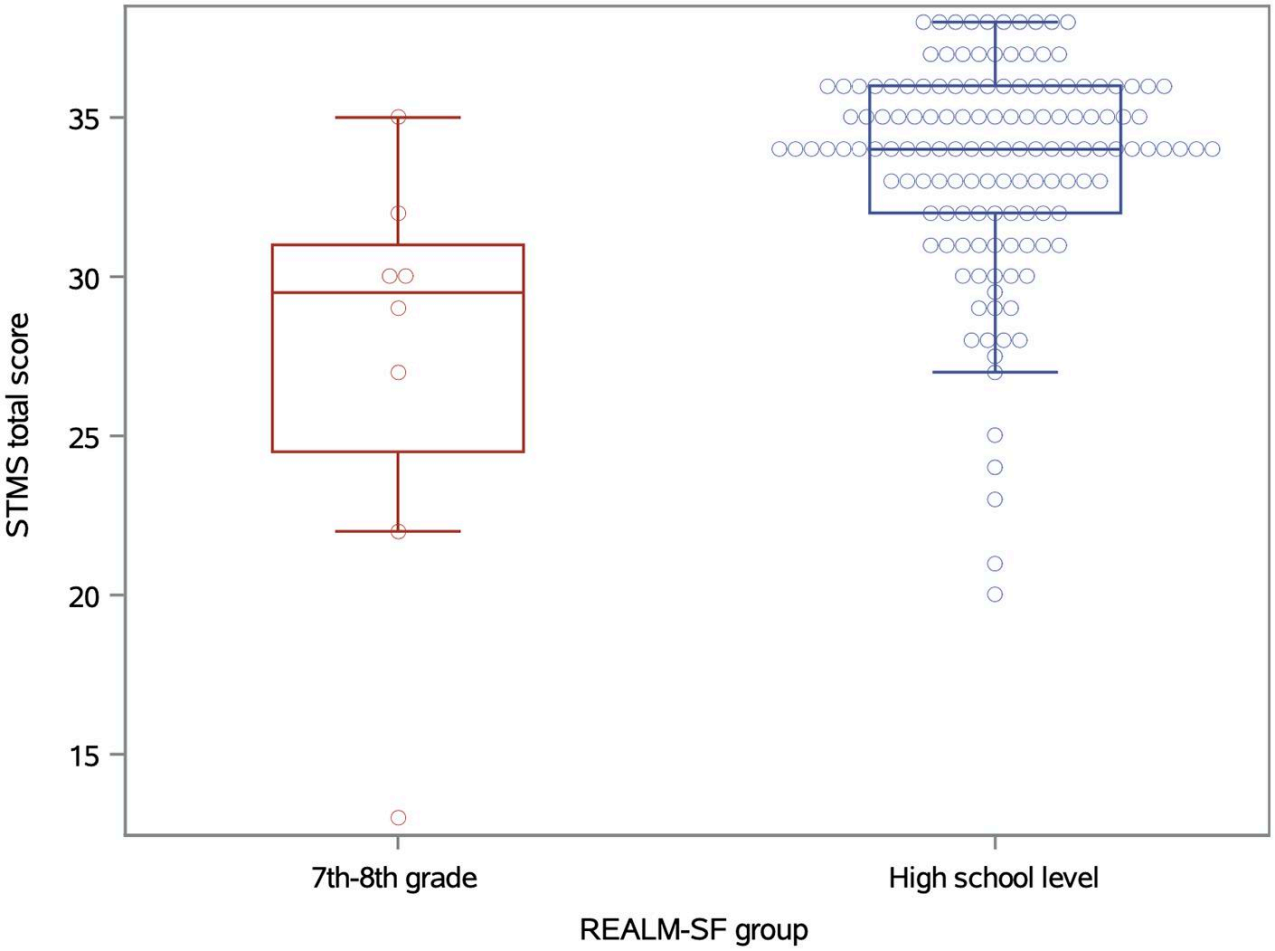
Boxplots of STMS scores across REALM-SF reading comprehension levels.

**Figure 2:**
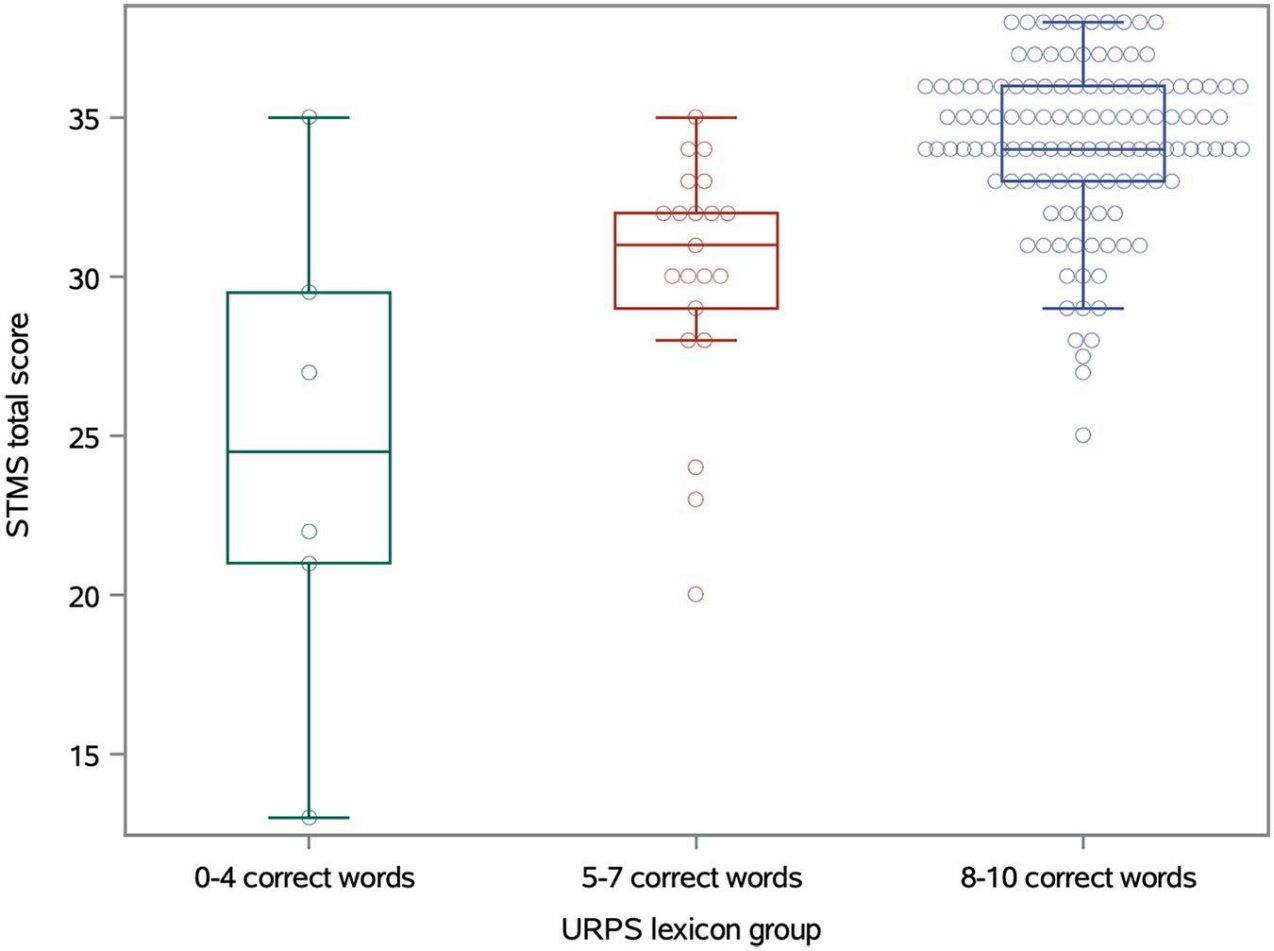
Boxplots of STMS scores across URPS lexicon groups.

Only eight patients (5%) scored in the 7th–8th grade range of health literacy as measured by the REALM-SF, of whom only one scored in the no impairment range of the STMS. These patients did have significantly lower STMS scores (mean = 27.3) as compared to the patients with high-school level health literacy (mean = 33.6; t-test *p* = 0.036; Figure 1). There is also a significant association between the STMS score and the number of Lexicon words identified, with a mean STMS score of 24.6 for patients identifying 0–4 Lexicon words, a mean STMS of 30.1 for patients identifying 5–7 words, and a mean of 34.2 for patients identifying 8–10 words (GLM *p* < 0.0001; Figure 2). The REALM-SF and the URPS Lexicon results were also significantly associated (Chi-square *p* < 0.0001). None of the patients with lower health literacy according to the REALM-SF were able to identify at least nine of the URPS Lexicon words, with one patient correctly matching eight of the Lexicon words. Conversely, 86% of the patients (120/140) with high school level health literacy by the REALM-SF were able to correctly identify at least eight of the Lexicon words.

## Discussion

This prospective blinded study in a URPS clinic at a tertiary care center was designed to explore patient-physician barriers that could affect patient management. We found that most patients had an understanding of most of the words in the Lexicon used in the URPS clinic, however, the words most commonly missed (vagina, bowel, pelvis, and urethra) are words that are very commonly used in the URPS clinic in particular. Although we do not know if these words were missed due to unfamiliarity or cultural background, a better patient understanding of these words could help facilitate patient counseling and shared decision-making. We also found that most patients had no cognitive impairment according to their STMS score, however, 40% of patients (*n* = 59) scored in the dementia or mild impairment range, of which only two were correctly identified as having cognitive impairment by the provider. This striking discordance between provider perception and screening-identified cognitive impairment may pose challenges for patient counseling, as unrecognized impairment can affect comprehension, recall, and adherence to care recommendations. This can hinder shared decision-making and impact patient health outcomes. The disparity between the provider's perception of patient health literacy and the results of the Lexicon vocabulary sheet also highlights the gap in provider-patient communication and the importance of educating providers on accurately identifying cognitive impairment and low health literacy. Simplifying terminology and providing glossaries during consultations may help address the barrier posed by low health literacy. Patient reluctance to ask clarifying questions can lead to incomplete understanding of treatment plans and suboptimal outcomes, so it is important to create a welcoming environment and encourage questions to improve patient understanding, engagement, and to facilitate shared decision-making. Trusted, accessible resources, such as patient-friendly handouts or reputable online resources tailored to urological conditions in the URPS clinic can further bridge the gap created by disparities in health literacy. Of note, there was not a significant difference found in age distribution between the STMS groups, so providers should refrain from using the patient's age as the sole determinant of cognitive status.

Several factors may contribute to underrecognition of cognitive impairment in specialty clinic settings. Patients with mild impairment may compensate during brief encounters, rely on rehearsed responses, or present with concerns that mask subtle deficits. Time constraints, competing clinical priorities, and lack of routine cognitive screening in subspecialty clinics may further limit detection during standard visits (11, 12).

Existing literature related to this topic usually focuses on either patient health literacy or cognitive impairment, rather than addressing both. There is scant literature on health literacy and cognitive impairment within urology, especially female benign urology, and existing literature on physician communication as it relates to patient health literacy does not specifically focus on the context of the urology clinic (mainly in primary care). The findings in this study corroborate a 2016 study conducted at a large tertiary care center for urology that demonstrated that a substantial portion of the patient population in urology has at least mild cognitive impairment (7). Our correlation analysis found significant overlaps in patients with low health literacy and cognitive impairment, which raises the question as to whether the lower literacy is affecting understanding of the STMS instructions, or if cognitive impairment is resulting in loss of health literacy performance. Although education and income level are also possible confounders to this analysis, the less varied demographics of this cohort precluded a formal multivariate analysis.

In everyday practice, urologists in the URPS clinic setting can help bridge the gap created by low health literacy by simplifying explanations or using visual aids. Creating a welcoming environment for patient questions, encouraging questions without judgment, and implementing cultural sensitivity can also help facilitate the patient-provider relationship. Strategies for simplifying communication may include using plain language and focusing on grouping information into chunks and breaking down explanations into manageable parts. Using the teach-back method, asking patients to repeat or illustrate their understanding of the information provided to ensure clarity, could also help facilitate patient understanding. The use of visual aids and resources in varying levels of readable formats in addition to multilingual resources might help facilitate patient understanding in the context of low health literacy and cognitive impairment. The leveraging of digital tools, videos, and reminder apps can aid in the explanation of conditions or procedures, medication instructions, and scheduling. Involving caregivers and family members as well as social workers or patient navigators as part of the patient's support system can be especially beneficial for patients with cognitive impairment. Clinical processes that could be improved include standardized assessments and screening tools, longer patient appointments (if possible), and more accessible clinic learning resources (i.e., pamphlets with pictures and larger text). Partnering with local organizations, libraries, and health literacy programs to provide materials tailored to this patient population can help ensure patients have access to education on their health even outside the clinic setting. It is important to continuously assess patient understanding and seek feedback on education materials and communication techniques to ensure the best methods remain effective.

This qualitative approach allowed for some insight into the provider perspective in their decision to consider a patient as having cognitive impairment or inadequate health literacy. This study also implemented standardized measures of health literacy and cognitive status that were specific for the context of the URPS clinic. Data collection took a relatively short amount of time, allowing for the collection to occur with the patient immediately before the visit with the physician and with the physician immediately after the visits, facilitating the validity of our comparisons without significantly affecting the normal workflow of the clinic.

Limitations of this study include potential biases in patient responses and lack of generalizability to the general population, as most patients were higher-income white women in a large tertiary care center. The city of Dallas also has a high Hispanic and Spanish-speaking population, so the exclusion of non-English speaking patients may have further biased patient demographics. Additionally, patients who were not at their first URPS visit may have had prior visits with a health care provider that would give them prior exposure to words listed in the Lexicon. Although the duration of the questionnaires was intended to be short, it is possible that fatigue or performance anxiety may have influenced the scores.

Future directions for this study could include expanding the study population. Reproducing this study in a different clinical setting (i.e., county hospital, rural setting) could provide insight into additional barriers to patient care. Additional work will be needed to determine if there is a way to recognize patients with cognitive impairment with more time-efficient measures, which now can be powered based on these preliminary findings.

## Conclusion

While most patients demonstrated adequate word recognition, the frequent misidentification of certain terms in the URPS Lexicon suggests gaps in understanding of commonly used medical terminology commonly used in this clinic setting. Discordance between provider assessments and standardized screening results highlight the need for improved recognition of cognitive impairment and adequate health literacy in specialty clinic settings. Addressing these gaps may improve communication, shared decision-making, and patient care.

## Data Availability

The raw data supporting the conclusions of this article will be made available by the authors, without undue reservation.
